# Discovery of Novel Orally Active Tetrahydro-Naphthyl-*N*-Acylhydrazones with *In Vivo* Anti-TNF-α Effect and Remarkable Anti-Inflammatory Properties

**DOI:** 10.1371/journal.pone.0156271

**Published:** 2016-05-26

**Authors:** Natália M. Cordeiro, Rosana H. C. N. Freitas, Carlos A. M. Fraga, Patricia D. Fernandes

**Affiliations:** 1 Federal University of Rio de Janeiro, Institute of Biomedical Science, Laboratory of Pharmacology of Pain and Inflammation, Rio de Janeiro, Brazil; 2 Federal University of Rio de Janeiro, Institute of Biomedical Science, Graduate Programm in Pharmacology and Medicinal Chemistry, Rio de Janeiro, Brazil; 3 Federal University of Rio de Janeiro, Institute of Biomedical Science, Laboratory of Evaluation and Synthesis of Bioactive Substances (LASSBio), Rio de Janeiro, Brazil; 4 Federal University of Rio de Janeiro, Chemistry Institute, Graduate Programm in Chemistry, Rio de Janeiro, Brazil; Federal University of Rio de Janeiro, BRAZIL

## Abstract

LASSBio-1524 was designed as inhibitor of the IKK-β (kappa β kinase inhibitor) enzyme, which participates in the activation of the nuclear factor κB (NF-κB) canonical pathway, and its three *N*-acylhydrazone new analogues, LASSBio-1760, LASSBio-1763 and LASSBio-1764 are now being tested on their anti-inflammatory potential. The activity of these compounds was evaluated with the subcutaneous air pouch induced by carrageenan and by subsequent measurement of tumor necrosis factor-α (TNF-α), nitric oxide (NO) and reactive oxygen species (ROS). In the acute inflammation model, the oral pretreatment with doses from 0.3 to 30 mg/kg of *N*-acylhydrazone derivatives was able to significantly reduce leukocyte migration to the cavity. Pretreatment with LASSBio-1524 and its analogues also decreased NO, TNF-α and ROS biosynthesis an events closely involved with NF-kB pathway. The tetrahydronaphthyl-*N*-acylhydrazone derivative LASSBio-1764 was the most promising compound from this series, surpassing even LASSBio-1524. Additionally, none of the compounds demonstrated myelotoxicity or cytotoxicity. Cell viability was assayed and these compounds demonstrated to be safe at different concentrations. Western blot analysis demonstrated that LASSBio-1524 and LASSBio-1760 inhibited NF-κB expression in RAW 264.7 cell lineage. Our data indicate that the tested compounds have anti-inflammatory activity, which may be related to inhibition of leukocyte migration, reducing the production of NO, TNF-α and ROS. LASSBio-1524 and LASSBio-1760, in addition to these features, also reduced p65 nuclear expression assessed by western blot in RAW 264.7 murine cells.

## Introduction

There are several pathways and molecular mediators that modulate survival and death of leukocytes at inflammation sites including the family of intracellular molecules of signaling pathways PI3K (phosphatidylinositol-3 kinase), cascade of MAPK (mitogen-activated protein kinase) and NF-κB (nuclear factor kappa B) [1, 2], pro-inflammatory cytokines [3] and reactive oxygen species (ROS) [4].

The transcription factor NF-κB has described action on several cells that make up the complex organisms, presenting a range of action exceeding all transcription factors previously characterized [5]. NF-κB has served as a model system for inducible transcription and due to their broad physiological and clinical effects, attracts great research interest. However, the role of NF-κB even raises questions about how a limited set of signaling mediator is able to integrate different stimuli to reach a specific cell type and response [6]. Various types of stimuli can activate the transcription factor NF-κB, such as viral, bacterial, inflammatory cytokines and antigen-receptor engagement, as well as mechanical and chemical stimuli, including the oxidative stress [7].

Under resting conditions, NF-κB dimers are linked to IκBs (κB inhibitory protein) sequestering the complexes of inactive NF-κB in the cytoplasm. An induced stimulus triggers the degradation of IκB proteins through phosphorylation by IκB kinase (IKK) complex, which consists of two catalytically active kinases, IKKα and IKKβ, and a regulatory subunit IKKγ (NEMO). Phosphorylated IκB is targeted for ubiquitination and proteosomal degradation, which leads to the release of NF-κB dimers that can translocate to the nucleus. Since activation depends on IκB degradation, the IKK complex is like the guardian of the NF-κB signaling and is critical for interaction with parallel signaling pathways [8].

There are some pathological conditions that characterize the involvement of NF-κB expression, i.e., inflammatory bowel disease (Chron’s disease and colitis), endometriosis and arthritis rheumatoid. These conditions are of difficult treatment and with several adverse effects. In this regard, the search of new drugs, more potent and with fewer toxic effects continues to be a goal.

**Fig 1:**
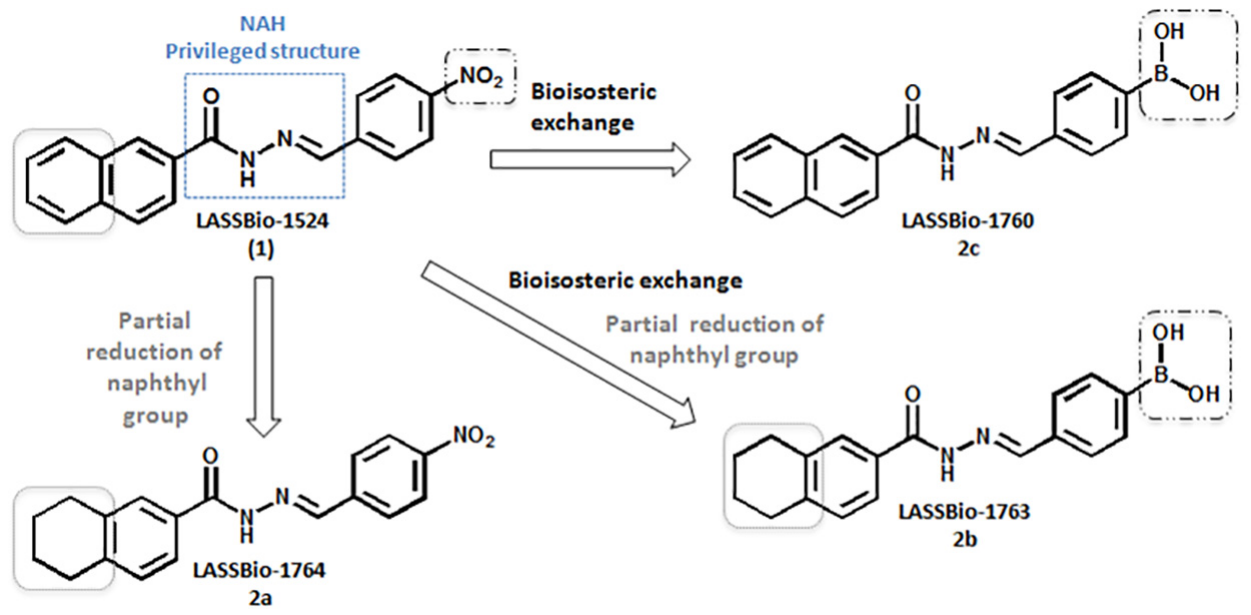
LASSBio-1524 and the design concept of the novel *N*-acylhydrazone derivatives (2a-c).

Previous work from our research group reported the discovery of *N*-acylhydrazone derivative LASSBio-1524 (substance 1, Fig 1) through structure-based drug design studies, as an IKKβ inhibitor with potent anti-inflammatory properties [9]. However, LASSBio-1524 needed to be administered intraperitoneally, since its poor aqueous solubility limited its administration by oral route and its development as drug candidate.

**Fig 2:**
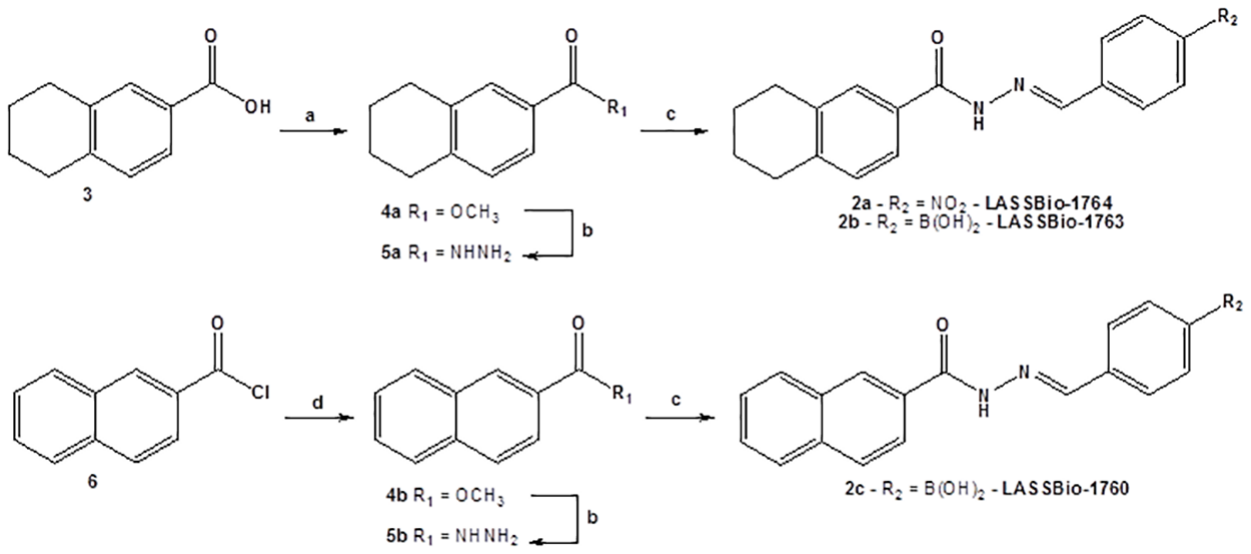
Synthesis of *N*-acylhydrazone derivatives 2a-c. Reagents and conditions: a) MeOH, H_2_SO_4 cat._ 70°C, 6 h, 90%; b) NH_2_NH_2_ H_2_O 80%, EtOH, 80°C, 4 h, 84–88%; c) ArCHO, EtOH, HCl 10%, 3 h, 75–94%; d) MeOH, r.t., 2 h, 86%.

In order to optimize the pharmacological activity of LASSBio-1524, we proposed three new *N*-acylhydrazone derivatives based on their structure. The *N*-acylhydrazone (NAH) framework was maintained in all compounds since it is recognized as a privileged structure [10] and changes were made in the aryl groups attached to acyl and imine subunits at NAH moiety. In LASSBio-1764 (substance 2a, Fig 1) and LASSBio-1763 (substance 2b, Fig 2) naphthyl ring of LASSBio-1524 was partially reduced in order to diminish the extension of planarity of the aromatic system and therefore try to improve the aqueous solubility profile, as previously described in literature [11]. LASSBio-1760 (2c) and LASSBio-1763 (2b) were proposed by isosteric exchange of the nitro group of LASSBio-1524 by a boronic acid, since this latter is able to perform more interactions with water, as H-bond acceptor and donor, and thus increase the aqueous solubility.

## Results and Discussion

### Chemistry

The synthesis of the new *N*-acylhydrazone derivatives (2a-c) was carried out as outlined in Fig 2. LASSBio-1763 (2a) and LASSBio-1764 (2c) were obtained by linear three steps synthesis. Commercially available 5,6,7,8-tetrahydronaphthalene-2-carboxylic acid (3) was submitted to reacted by Fischer esterification condition by treatment with methanol and catalytic sulfuric acid at reflux to furnish the corresponding methyl ester (4a). Hydrazides (5a or 5b) were obtained by reaction from methyl ether (4a or 4b) and hydrazine hydrate 80% in ethanol at reflux [12]. Finally, hydrazide (5a) is condensed with the appropriate aromatic aldehydes under acid catalysis at room temperature to generate LASSBio-1763 (2a) and LASSBio-1764 (2c) in high yields [12]. For the synthesis of LASSBio-1760 (2c), the starting material was the commercially available 2-naphthoyl chloride (6), which was treated with methanol at room temperature to give the corresponding methyl ester (4b). Acid-catalyzed condensation of the 2-naphthohydrazide (5b) and 4-formylphenylboronic acid resulted in LASSBio-1760 (2c) [12]. Yields, purity and physical properties of *N*-acylhydrazone derivatives 2a-c are shown in Table 1.

**Table 1:** Yields and physical properties of *N*-acylhydrazone derivatives 2a-c.

Compound	Molecular Formula	Purity (%)^a^	Yield (%)^b^	m.p. (°C)
LASSBio-1764 (2a)	C_18_H_17_N_3_O_3_	99.252	94	226–228
LASSBio-1763 (2b)	C_18_H_19_BN_2_O_3_	95.917	91	222–224
LASSBio-1760 (2c)	C_18_H_15_BN_2_O_3_	98.187	75	240–242

^a^Obtained by high performance liquid chromatography (HLPC)

^b^Yields refer to the condensation step of hydrazides 4a–b with the corresponding aromatic aldehydes.

**Fig 3:**
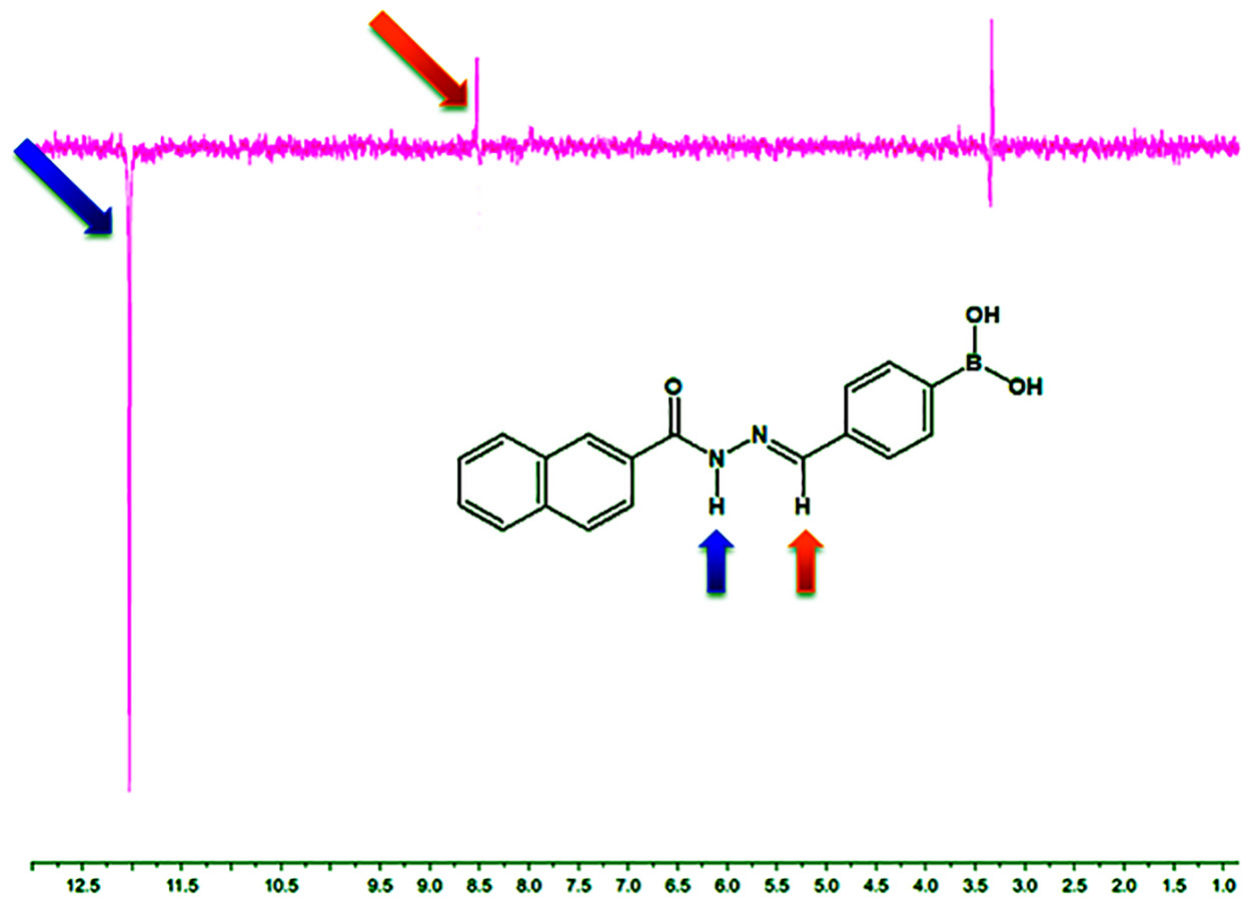
NOESY experiment of N-acylhydrazone derivative LASSBio-1760 (2c).

The synthesis of LASSBio-1760 (2c) was confirmed by analysis of ^1^H-NMR spectra, where was observed the presence of characteristic signals of the NAH function, the singlet at 12.08 ppm, referring to the amide hydrogen and the singlet at 8.55 ppm (Fig 3), which corresponds to the imine hydrogen. The absence of duplicity on the signals related to imine and amide hydrogens of NAH subunit is a strong indicative of the presence of only one diastereomer, probably presenting the relative (*E*)-configuration. In addition, these results are consistent with other published works of our research group describing the relative configuration of *N*-acylhydrazones [13, 14]. However, in order to obtain a further indication that the diastereomer obtained exhibits the relative (*E*)-configuration, we carried out the NOESY (spectroscopy nuclear Overhauser effect) experiment on derivative LASSBio-1760. Thus, the amide hydrogen was irradiated and the influence was observed on the hydrogens spatially closer to it, such as the imino hydrogen of the (*E*)-diastereomer. In fact, the NOESY experiment confirmed that the amide hydrogen is spatially near to the imine hydrogen, since we were able to detect nuclear Overhauser effect (NOE) (Fig 3) between them. This finding is an additional evidence that only the (*E*)-diastereomer was formed, once that the (*Z*)-diastereomer does not have the same spatial proximity between the amide and imine hydrogens. LASSBio-1763 and LASSBio-1764 also had their structure confirmed by ^1^H-NMR analysis, which also indicated the presence of the characteristic signals of the hydrogens at NAH moiety in approximately 12 and 8.5 ppm. Both signals showed no duplicity, probably indicating the presence only of the (*E*)-diastereomer.

Unfortunately, despite the design concept of the new *N*-acylhydrazone derivatives 2a-c are focused in the optimization of some drug-like properties, the evaluation of its aqueous solubility profile indicated that this parameter did not change significantly. LASSBio-1763 (2b) and LASSBio-1764 (2a) showed minimal improvement in the solubility profile (Table 2), while LASSBio-1760 (2c) showed a worsening in the aqueous solubility when compared to LASSBio-1524 (1). Thus, one way to minimize the problem of low solubility of compounds was used polysorbate 80 as vehicle. This allowed to administer all *N*-acylhydrazone derivatives orally, including LASSBio-1524.

**Table 2:** Aqueous solubility and other drug-like properties of *N*-acylhydrazone derivatives (2a-c).

Compound	Aqueous Solubility(mg/mL)^a^	*c*Log P^b^	PSA (Å^2^)^c^
LASSBio-1524 (1)	1.2 x 10^−3^	3.56	73.090
LASSBio-1764 (2a)	1.0 x 10^−3^	4.04	73.119
LASSBio-1763 (2b)	1.5 x 10^−3^	3.27	71.768
LASSBio-1760 (2c)	4.4 x 10^−4^	2.98	71.340

^a^ Solubility was determined by ultraviolet spectroscopy as described by Schneider and co-workers [15].

^b^ *c*Log P was calculated using program ACD Percepta.

^c^ PSA (polar surface area) was calculated using program Spartan, Wavefunction, Inc.

### Pharmacology

#### Anti-inflammatory effect in the subcutaneous air pouch model (SAP)

Acute inflammation induced by carrageenan is characterized by accentuated cell migration, predominance of polymorphonuclear cells, increased vascular permeability, extravasation of plasma proteins and increased levels of inflammatory mediators, such as nitric oxide and cytokines [15–18]. The first model used to investigate the anti-inflammatory potential of new NAH derivatives was the subcutaneous air pouch model (SAP). This model can be used for the wide quantitative analysis of the mediators involved in the inflammatory process, which makes it an excellent tool to study and screening new anti-inflammatory agents [19]. Additionally, it is worth noting that Ellis and colleagues [20] reported that this model involves the activation of NF-κB, thus highlighting the role of this transcription factor in induced inflammation.

**Fig 4:**
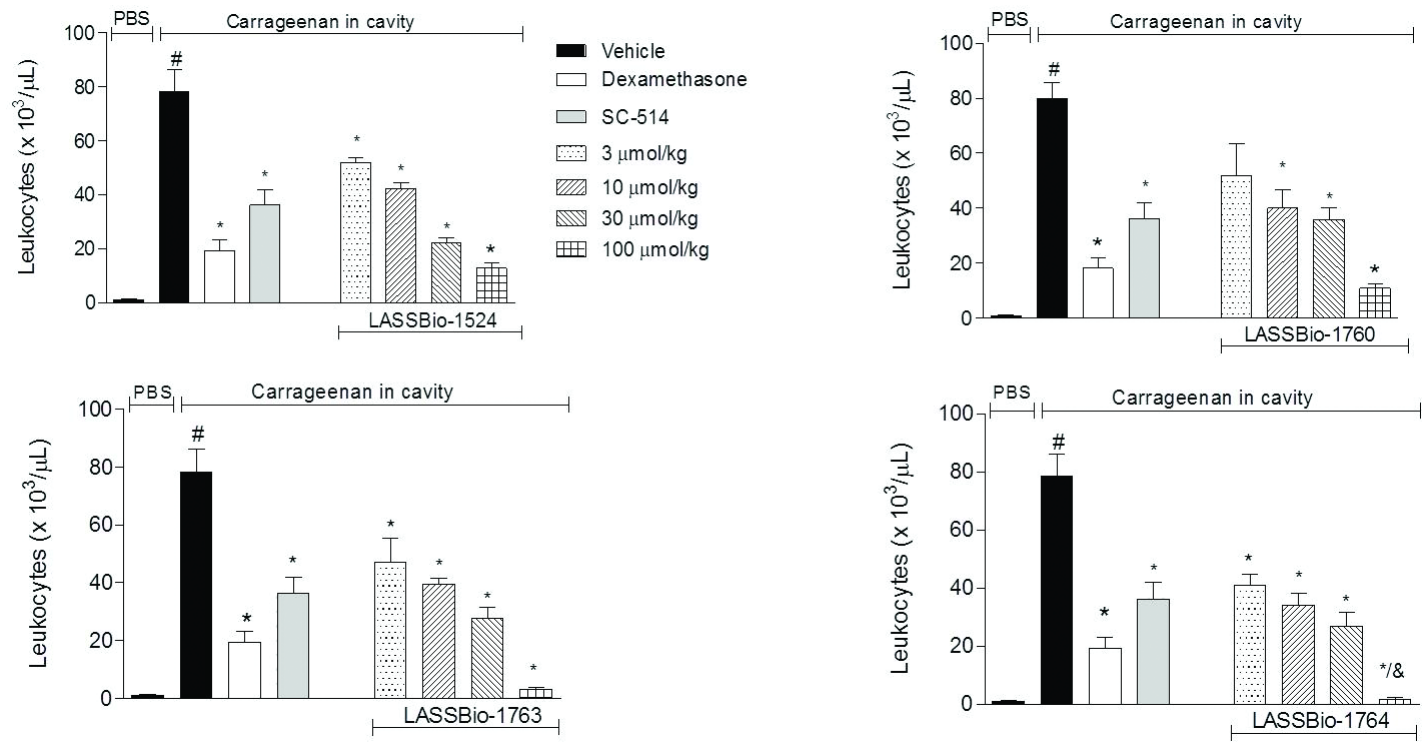
Effect of *N*-acylhydrazone derivatives in leukocyte migration induced by carrageenan in subcutaneous air pouch (SAP). The animals were pretreated orally 60 minutes before carrageenan injection in the SAP, with vehicle (Polysorbate 80) or compounds at doses of 0.3, 3, 10 or 30 mg/kg, IKK-β inhibitor SC-514 (10 mg/kg) or intraperitoneally pretreated with dexamethasone (2.5 mg/kg). Results were expressed as mean ± standard deviation of the number of total leukocytes (x 10^3^/μL). Statistical significance (p <0.05) was calculated by analysis of variance (ANOVA) followed by Bonferroni post-test. ^#^ p<0.05 when compared to the vehicle-treated animals in the group of PBS in SAP; *p<0.05 compared to the vehicle-treated animals in the group of carrageenan in the SAP.

The IKK inhibitor SC-514 (S1 Fig) and dexamethasone were chosen as standards for this model. Our results indicate that all new compounds (**2a**-**c**) and LASSBio-1524 (**1**) reduced significantly influx of leukocytes that migrate to the cavity at all doses tested, showing a dose-response (Fig 4).

LASSBio-1764 (2a) and LASSBio-1763 (2b) were the most promising compounds in this model, since they were able to decrease dramatically leukocytes into the air pouch at 30 mg/kg, 98% and 96%, respectively. This is an indicative that the strategy of reducing partially the naphthyl subunit of LASSBio-1524 (1) was great to increase the anti-inflammatory activity.

At the highest concentration tested, all new compounds (2a-c) were better than the prototype LASSBio-1524 (1), being more effective in reducing cell migration. Complementarily, at the concentration 10 mg/kg, all new compounds (2a-c) and LASSBio-1524 (1) exhibited an activity similar to that of IKKβ inhibitor at the same concentration. This may be an indication that the anti-inflammatory action of the new compounds is mediated by blocking IKKβ-NF-κB pathway.

Interestingly, the reduction of the aromatic ring of naphthyl proved to be better for anti-inflammatory activity, as LASSBio-1763 (2b) and LASSBio-1764 (2a) were better than the corresponding analogues with the naphthyl group, LASSBio-1524 (1) and LASSBio-1760 (2c). Additionally, the nitro group demonstrated contributes more to the anti-inflammatory action than the boronic acid group. However, since the compounds exhibit ED_50_ nearby for this activity, it can be said that 4-nitrophenyl subunit has bioisosteric relationship with the 4-phenyl boronic acid subunit.

LASSBio-1764 (2a) was the compound that showed the best activity and, consequently showed the best ED_50_ among the tested compounds, *i*.*e*. 1.5 mg/kg (Table 3). Furthermore, it is important to note that again LASSBio-1764 was better than LASSBio-1524 prototype (1), who presented ED_50_ 2.7 mg/kg. LASSBio-1763 (2b) also showed similar ED_50_ value to LASSBio-1764, *i*.*e*. 2.1 mg/kg.

**Table 3:** ED_50_ values of the N-acylhydrazone derivatives LASSBio-1760 (2c), LASSBio-1763 (2b) and LASSBio-1764 (2a) and the LASSBio-1524 (1) in the subcutaneous air pouch (SAP) model in mice, after oral administration.

N-acylhydrazone derivative	ED_50_
LASSBio-1524 (1)	2.7 mg/kg
LASSBio-1764 (2A)	1.5 mg/kg
LASSBio-1763 (2b)	2.1 mg/kg
LASSBio-1760 (2C)	4.8 mg/kg

The active form of NF-κB is already found in the SAP model from the first day of the experiment [20]. Thus, the action of NAH compounds by blocking mediators that control NF-κB activation pathway or the nuclear factor itself would explain the significant anti-inflammatory activity and evidenced a reduction of cytokines precisely whose expression is regulated by this transcription factor.

#### Decrease of Nitric oxide (NO) production in vivo

**Fig 5:**
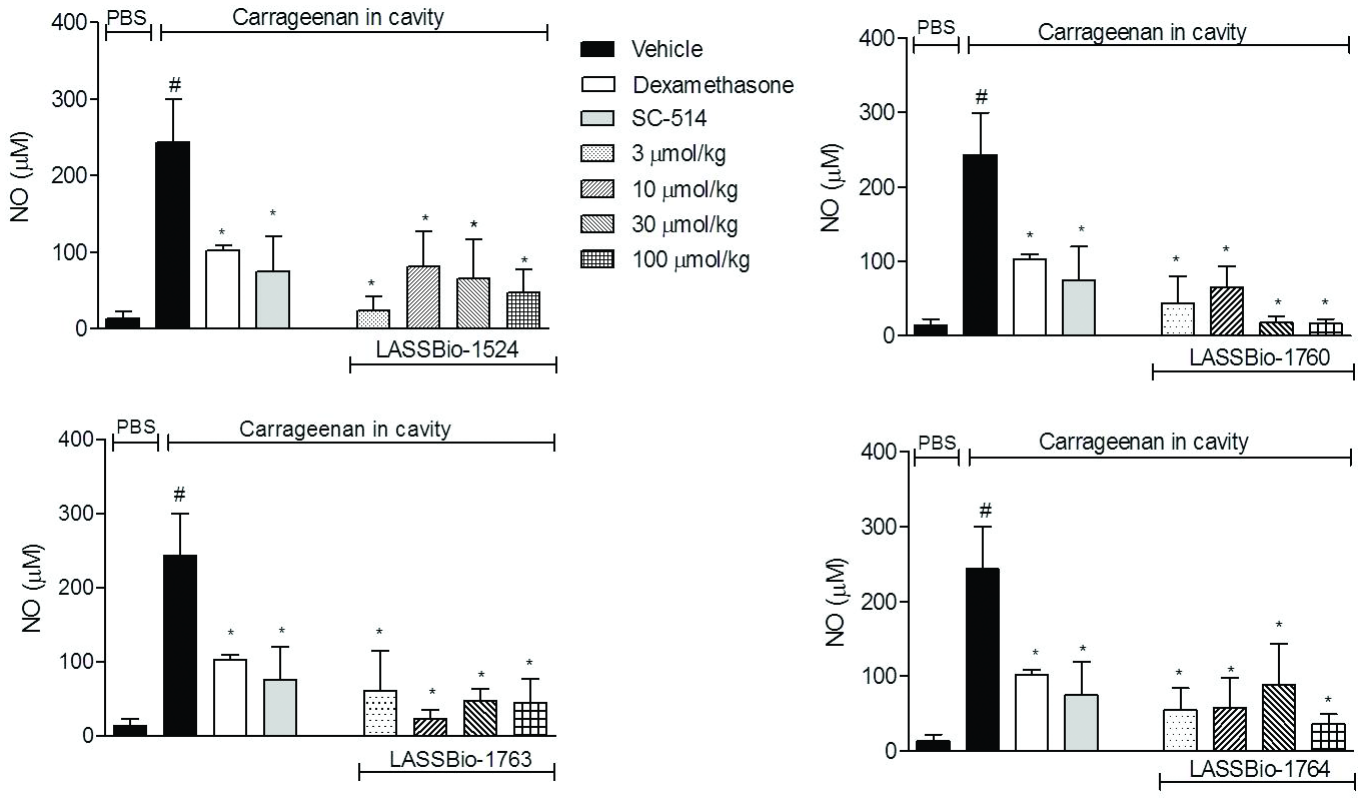
Effect of *N*-acylhydrazone derivatives in nitric oxide production induced by carrageenan at SAP. The animals were pretreated orally 60 minutes before carrageenan injection in the SAP, with vehicle (Polysorbate 80) or *N*-acylhydrazone derivatives at doses of 0.3, 3, 10 or 30 mg/kg, IKK-β inhibitor SC-514 (10 mg /kg) or intraperitoneally pretreated with dexamethasone (2.5 mg /kg). Results were expressed as mean ± standard deviation of the number of total leukocytes (x 10³/μL). Statistical significance (p <0.05) was calculated by analysis of variance (ANOVA) followed by Bonferroni post-test. ^#^ p<0.05 when compared to the vehicle-treated animals in the group of PBS in SAP; *p<0.05 compared to the vehicle-treated animals in the group of carrageenan in the SAP.

In order to access inflammatory mediators that are produced during the inflammatory process and a possible effect of the substances, the amount of nitric oxide (NO) accumulated in the SAP exudate was quantified. As shown in Fig 5, animals pretreated orally with vehicle and injected with PBS in SAP, had a concentration of 13.4 ± 8.6 μM of NO, while in animals pretreated orally with vehicle and that received carrageenan injection in SAP the NO production increased almost 19-fold (243.3 ± 56.5 μM). Pretreatment with SC-514 or dexamethasone reduced in 70% and 58% NO levels (74.8 ± 44.8 μM and 102.4 ± 6.3 μM, respectively). Our results also indicate that pretreatment with all *N*-acylhydrazone derivatives reduced by more than 65% NO production. At the lowest dose, 0.3 mg/kg, the reduction was almost equal to the SC-514 treated group (Fig 5). This result is another evidence that the compounds are perhaps acting by blocking the IKKβ-NF-κB pathway.

In addition, LASSBio-1760 (2c) almost completely blocked NO production in doses of 10 and 30 mg/kg, 94% and 93%, respectively. This suggests that these substances could be acting through direct inhibition of nitric oxide synthase (iNOS), reducing its enzymatic activity or perhaps inhibiting the biosynthesis/iNOS expression in cells [21–23].

We also confirmed that compounds affects chemotaxis through the inhibition of NO production and pro-inflammatory cytokines levels resulting in the suppression of inflammatory process. It is interesting to note that these inhibitory properties were similar with the anti-inflammatory effects produced by dexamethasone [19]. This steroidal anti-inflammatory drug is effective in inhibiting cell migration, NO production and cytokines levels, as well as leukocytes influx.

Blockade of NO has the potential to deliver therapeutic benefit in inflammation, once its overproduction is present in a number of diseases such as arthritis, inflammatory bowel disease, Alzheimer's, Parkinson's and others [24, 25]. It has been observed that blockade of NO in acute models of inflammation contributes to the improved reduction of the inflammatory phenomena [26, 27].

#### Extensive inhibition of TNF-α production

**Fig 6:**
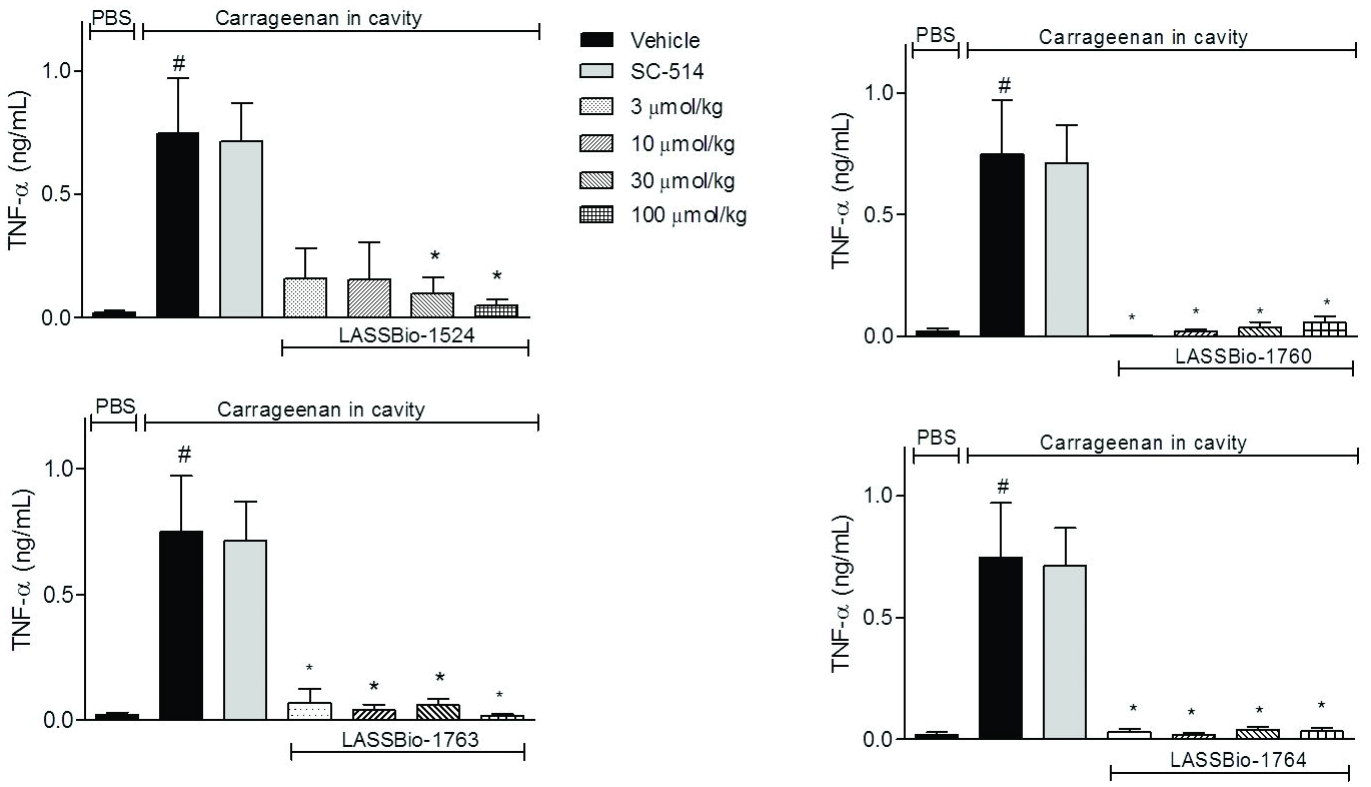
Effect of *N*-acylhydrazone derivatives in TNF-α production induced by carrageenan at SAP. The animals were pretreated orally 60 minutes before carrageenan injection in the SAP, with vehicle (Polysorbate 80) or *N*-acylhydrazone derivatives at doses of 0.3, 3, 10 or 30 mg/kg, IKK-β inhibitor SC-514 (10 mg /kg) or intraperitoneally pretreated with dexamethasone (2.5 mg /kg). Results were expressed as mean ± standard deviation of the number of total leukocytes (x 10^3^/μL). Statistical significance (p <0.05) was calculated by analysis of variance (ANOVA) followed by Bonferroni post-test. ^#^ p<0.05 when compared to the vehicle-treated animals in the group of PBS in SAP; *p<0.05 compared to the vehicle-treated animals in the group of carrageenan in the SAP.

TNF-α level was quantified to evaluate if the reduction in cell migration would reflect only the number of cells or if there was interference of the transcription factor after treatment with the compounds. An increase in the production of this cytokine was evident in animals that received oral pretreatment with vehicle and SAP injection of carrageenan. The *N*-acylhydrazone derivatives 2a-c were able to significantly reduce levels of TNF-α. In all doses, new *N*-acylhydrazone derivatives (2a-c) almost abolished TNF-α levels, while LASSBio-1524 (1) reduced with less effectively the cytokine levels (Fig 6). Again, LASSBio-1763 (2b) and LASSBio-1764 (2a) were the most promising compounds and show a bioisosteric relationship for this pharmacological action.

TNF-α levels were reduced in, at least, 95%, at dose of 30 mg/kg, to all N-acylhydrazone derivatives. These data, together with those obtained by the inhibition of NO indicates that TNF-α and NO pathways are interconnected. Pro-inflammatory cytokines as TNF-α, trigger a series of signaling pathways, including the pathway of NF-κB and its level is known to be a direct correlation to the iNOS enzyme [28–30].

#### Decrease in production of reactive oxygen species (ROS)

**Fig 7:**
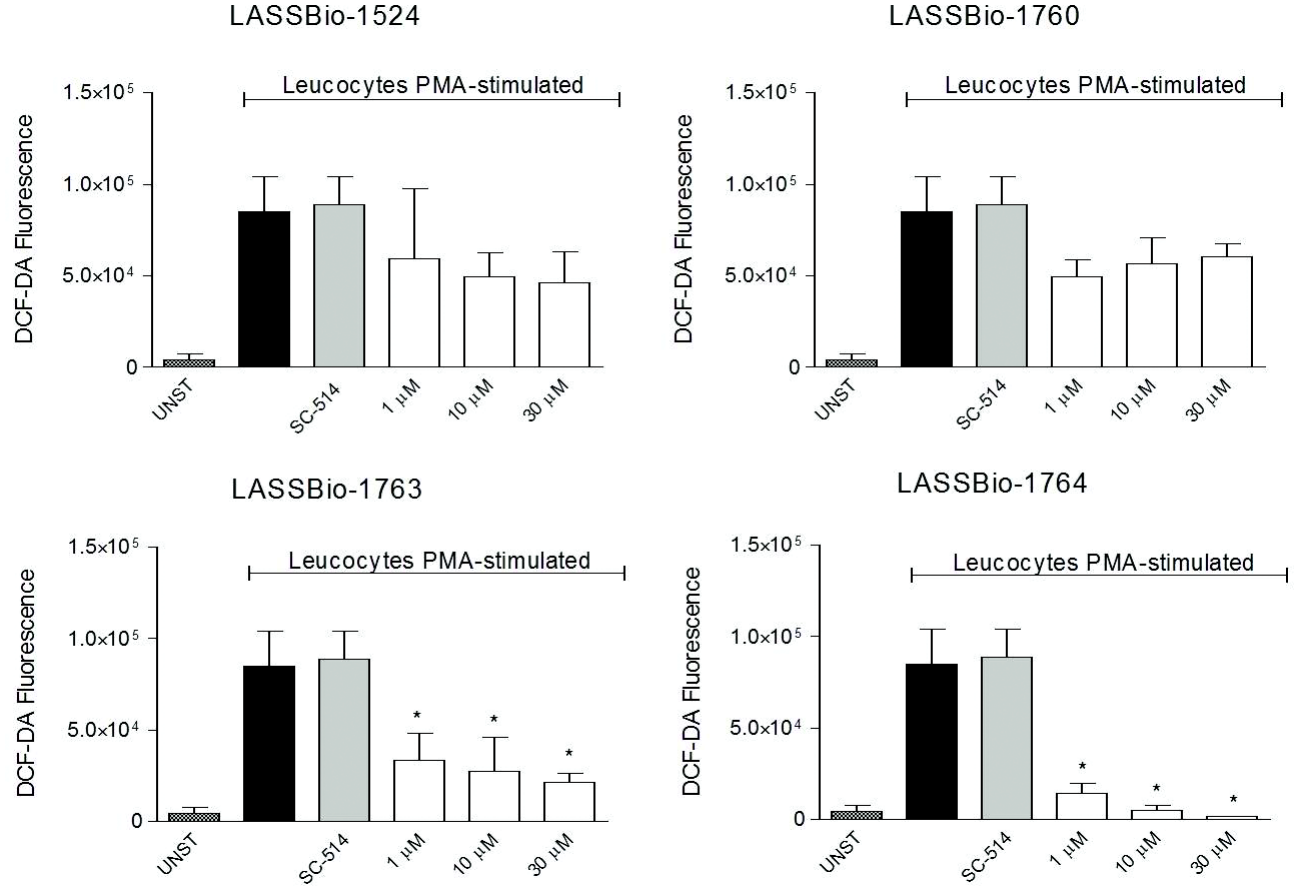
Oxidative metabolism of leukocytes collected from the SAP 24 hours after carrageenan injection, stimulated with PMA. Intracellular ROS levels were quantified and reading was performed by flow cytometry counting 10,000 events. DCF expression is presented in values of the geometric mean fluorescence intensity in FL1.

Fig 7 shows that only LASSBio-1763 (2b) and LASSBio-1764 (2a) significantly reduced the production of reactive oxygen species (ROS) by PMA-stimulated leukocytes. And the reduction caused by LASSBio-1764 (2a) almost abolished levels of ROS. Unfortunately, LASSBio-1760 (2c) and LASSBio-1524 (1) were not significantly active in this test, as the IKK inhibitor. However, it is possible to look again the bioisosteric relationship between boronic acid group and nitro group viewed by similar activity of LASSBio-1763 and LASSBio-1764 in reducing ROS biosynthesis.

Since oxidative stress may be harmful in the resolution of inflammation and the overproduction of reactive oxygen species (ROS) is the mechanism behind many neuropathologies, any impaired induction of antioxidant enzymes or antioxidants that are induced by NF-κB under normal conditions might be responsible for ROS accumulation. Studies demonstrated that antioxidant enzyme genes including manganese-dependent SOD (MnSOD), glutathione S-transferase, and ferritin heavy chain (FHC), are induced by TNF-α activation of NF-κB [31]. Our results indicate that inhibition in the production of ROS also appears to be part of the mechanism of action of tested N-acylhydrazone derivatives (1, 2a-c).

Since LASSBio-1524 is an IKKβ inhibitor, in order to check the inhibitory profile of its new analogs in the target enzyme, we performed an in vitro test in the CEREP Company. However, the new analogues, LASSBio-1760, LASSBio-1763 and LASSBio-1764 did not inhibit the enzyme activity in that protocol [32] until the concentration of 30 μM (Data not shown).

#### LASSBio-1524 and LASSBio-1760 attenuates the LPS-induced NF-kB expression in RAW 264.7

**Fig 8:**
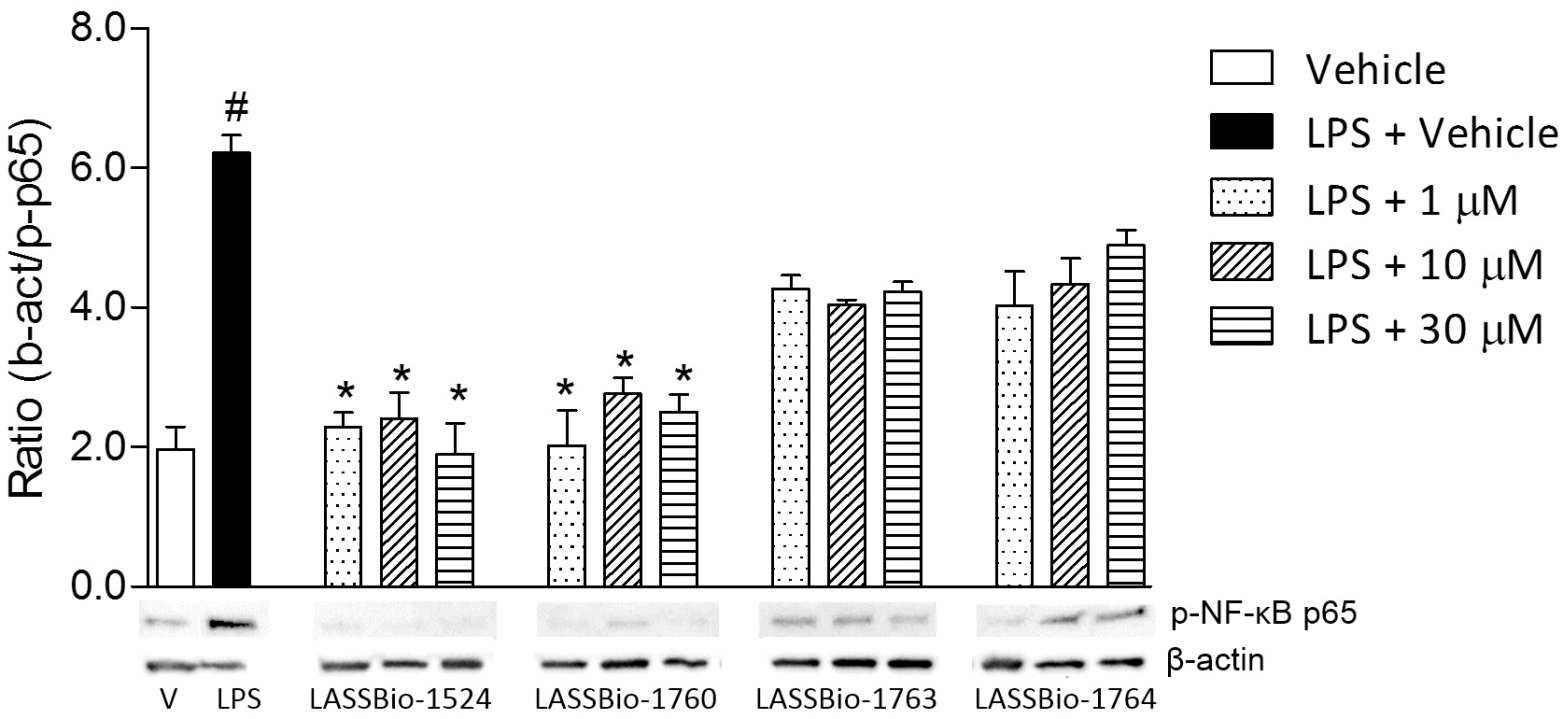
Cell lysates were prepared and Western blots were probed for phospho-NFkB p65 antibody. Western blot and densitometric analysis of NF-κB activation in LPS stimulated RAW 264.7 cells. Cells were stimulated with LPS (1 μg/mL) for 2 hours. β-actin protein levels were used as loading control. Representative Western blot of β-actin and phospho-NF-κB p65 (Ser536) expression. Equal amounts of nuclear proteins were loaded onto each lane. Graphical densitometry representation of ratio between β-actin and phospho-NF-κB p65 levels after incubation with 1, 10 or 30 μM for 2 hours. Negative control without LPS treatment. Each value is expressed as mean± SD of triplicate experiments. Statistical significance (*p <0.05) was calculated by analysis of variance (ANOVA) followed by Bonferroni post-test. Each Fig is representative of 3 independent experiments.

Since the analogues were not active in CEREP’s protocol, in order to determine a possible target for the anti-inflammatory action observed, we evaluated NF-κB p65 expression by western blot. NF-kB expression in RAW 264.7 murine cells was decreased with concentrations of LASSBio-1524 and LASSBio-1760 ranging from 1 μM to 30 μM (Fig 8). The compounds were also tested in the cells without LPS stimulation and demonstrated no activity per se (data not shown). However, did not significantly affect activation of NF-kb as assessed by kinase activity or western blot analysis, suggesting that they are not specific inhibitors of pospho p65 although in vivo LASSBio-1764 had a significant anti-inflammatory effect, the phosphorylation occurred normally and its potencial activity may be due to its effect on other mediators, as ROS, NO and TNF-α.

#### Myelotoxicity and Cytotoxicity Evaluation

Finally, to make sure that the inhibitory effect on cell migration was due to a direct effect on cellularity and not a nonspecific toxic effect, the leukocyte counting was performed in the blood and bone marrow of animals that were pre-treated with the higher dose (30 mg/kg) of each *N*-acylhydrazone derivatives. The results did not show any significant differences regarding the number of total leukocytes from the blood and bone marrow between the pretreated groups with the *N*-acylhydrazone derivatives (1, 2a-c) or control groups (S2 Fig). Cell viability was measured by the MTT assay [33] and the compounds did not reduce the cell viability of RAW 264.7 mouse macrophages at concentrations of up to 30 μM (S3 Fig). These data are a strong indication of the low toxicity of the compounds, showing their potential as drug candidates.

### Conclusions

We described the design and synthesis of novel *N*-acylhydrazone derivatives structurally analogues to LASSBio-1524 (1). Our results indicate that all compounds (2a-c) demonstrated a potential and significant anti-inflammatory activity, reducing leukocytes migration, TNF-α levels, NO production, inhibition of NF-kB enzyme expression and provided strong inhibition of free radical activities in in vivo assays. And it is worth pointing out that these *N*-acylhydrazone compounds were administered orally and show no myelotoxicity toxicity. In addition, a bioisosteric relationship between boronic acid and nitro subunits is clearly. The partial reduction of the naphthyl group of LASSBio-1524 (1) proved to be an extremely beneficial modification to the anti-inflammatory activity. LASSBio-1764 (2a) demonstrated to be the most promising compounds from this series, surpassing even LASSBio-1524 (1).

It seems that LASSBio-1760 inhibits NF-κB enzyme expression thus reducing production of final mediators dependent of this pathway. The mechanism of action remains to be elucidated, but a possible target may be the NF-κB since this is present in second messenger signaling pathways of NO, ROS and TNF-α, whose levels have been reduced. These findings provide evidence that supports these compounds as candidates to the treatment of several inflammatory diseases.

## Materials and Methods

### Chemistry

Reagents and solvents were purchased from commercial suppliers and used as received. The ^1^H and ^13^C NMR spectra were recorded on a Bruker AC-200 NMR, operating at 200 MHz for ^1^H and 50 MHz for ^13^C NMR. Thin layer chromatography (TLC) was performed on Merck KieselGel 60 F254 (0.2 mm thickness) using as the mobile phase n-hexane and ethyl acetate or dichloromethane and methanol at various concentrations. The TLC plates with the reactants and products were visualized with ultraviolet light at wavelengths of 254 and 365 nm.

Infrared spectra (IR) were carried out in the spectrophotometer apparatus Fourier transform IR Nicolet 6700 FT-IR using tablets of potassium bromide (KBr). Melting points were determined in Quimis Q340.23 apparatus and are uncorrected.

The purity of the final product was determined by High Performance Liquid Chromatography (HPLC) on Shimadzu LC-20AD with Kromasil 100–5 C18 column (4.6 mm x 250 mm) and Detector SPD-M20A (Diode Array). The quantification of analyte was performed using a standardized wave length, 308 nm. The mobile phase used was acetonitrile and water 60–80%, running time (R_t_) varying between 10 and 20 minutes.

The determination of the solubility in water was performed using UV-VIS spectrophotometer with Femto Scan Model 800XI.

#### Methyl 5,6,7,8-tetrahydronaphthalene-2-carboxylate (4b)

A solution of 0.5 g (2.83 mmol) of 5,6,7,8-tetrahydronaphthalene-2-carboxylic acid (3) in 30 mL of methanol was refluxed at 70°C for 6 hours. Afterwards, the solvent was partially concentrated at reduced pressure and the resulting mixture was extracted with dichloromethane (80 mL x 2) and brine. The organic layer was dried with anhydrous sodium sulfate and the solvent was evaporated to give 0,460 g of colorless oil. ^1^H-NMR (200 MHz, DMSO-*d*_*6*_): δ = 7.63 (2H, m); 7.16 (1H, d, *J* = 6 Hz); 3.84 (3H, s); 2.76 (4H, ls); 1.73 (4H, ls) [S4 Fig]. ^13^C- NMR (50 MHz, DMSO-*d*_*6*_): δ 166.3; 142.6; 137.1; 129.7; 129.2; 126.7; 126.0; 51.8; 28.8; 28.5; 22.3; 22.2 (S5 Fig). IR (KBr) cm^-1^: 1741 (ʋ C = O).

#### Methyl 2-naphthoate (4a)

A solution of 0.2 g (1.05 mmol) of 2-naphthoyl chloride (6), in 20 mL of methanol was stirred at room temperature for 2 hours. Afterwards, the solvent was partially concentrated at reduced pressure and the resulting mixture was poured into ice and cold water. The precipitate formed was filtered and dried under vacuum, producing the solid white powder in 86% of yield. ^1^H (400 MHz, CDCl_3_): δ 3.99 (3H, s); 7.53–7.61 (2H, m); 7.88 (2H, m); 7.95 (1H, d, *J* = 8 Hz); 8.05 (1H, d, *J* = 8 Hz); 8.62 (1H, d). ^13^C (50 MHz, DMSO-*d*_*6*_): δ 53.08; 125.44; 127.53; 127.87; 128.41; 129.16; 129.48; 129.95; 131.21; 132.69; 135.75; 167.55. IR (KBr): cm^-1^ 1713 (ʋ C = O).

#### General procedure for the preparation of 2-naphthohydrazide (5a) and 5,6,7,8-tetrahydronaphthalene-2-carbohydrazide (5b)

To a solution of 2.47 mmol (1 eq.) of methyl 2-naphthoate (4b) or methyl 5,6,7,8-tetrahydronaphthalene-2-carboxylate (4a) in absolute EtOH (20 mL) was added 7.73 mL (0.15 mmol, 40 eq.) hydrazine hydrate 80%. The mixture was refluxed at 80°C for 4 hours. Afterwards, solvent was partially concentrated at reduced pressure and the resulting mixture was poured into ice. The precipitate formed was filtered out and dried under vacuum, producing the desired product.

#### 5,6,7,8-tetrahydronaphthalene-2-carbohydrazide (5a)

White powder, 84%, ^1^H-NMR (200 MHz, DMSO-*d*_*6*_): δ = 1.77 (4H, ls); 2.76 (4H, ls,); 3.74 (2H, s); 7.06 (1H, d, *J* = 6 Hz); 7.42 (2H, m); 7.99 (1H, s) [S6 Fig]. ^13^C-NMR (50 MHz, DMSO-*d*_*6*_): δ 22.5; 28.7; 123.9; 127.6; 128.8; 130.4; 136.5; 140.0; 166.1 (S7 Fig). IR (KBr) cm^-1^: 1662 (ʋ C = O); 3200 e 3300 (ʋ N-H).

#### 2-naphthohydrazide (5b)

White powder, 88%, ^1^H-NMR (200 MHz, DMSO-*d*_*6*_): δ 4.59 (2H, s); 7.56–7.60 (2H, m); 7.94–8.00 (4H, m); 8.44 (1H, s); 9.94 (1H, s). ^13^C-NMR (50 MHz, DMSO-*d*_*6*_): δ 123.8; 126.6; 127.2; 127.4; 127.5; 127.8; 128.7; 130.6; 132.1; 134.0; 165.9. DEPT-135 (50 MHz, DMSO-*d6*): 124.3; 127.1; 127.7; 127.9; 128.0; 128.3; 129.2. IR (KBr) cm^-1^: 1619 (ʋ C = O); 3182 e 3314 (ʋ N-H).

#### General procedure for the preparation of N-acylhydrazone derivatives (2a-c)

To a solution of 1.61 mmol (1 eq.) of 2-naphthohydrazide (8) or 5,6,7,8-tetrahydronaphthalene-2-carbohydrazide (5) in absolute EtOH (20 mL) containing two drops of solution hydrochloric acid 10%, was added 1.7 mmol (1 eq.) of the corresponding aromatic aldehyde. The mixture was stirred at room temperature for 3 hours. Afterwards, the solvent was partially concentrated at reduced pressure and the resulting mixture was poured into ice. The precipitate formed was filtered out and dried under vacuum, producing the desired *N*-acylhydrazone in high yield.

#### (*E*)-*N*-(4-nitrobenzylidene)-5,6,7,8-tetrahydronaphthalene-2-carbohydrazide (2a, LASSBio-1764)

Beige powder purified by recrystallized from ethanol, 94%, ^1^H-NMR (200 MHz, DMSO-*d*_*6*_): δ 1.76 (4H, sl); 2.78 (4H, sl); 7.21 (1H, d, *J* = 8 Hz); 7.64 (2H, sl); 8.00 (2H, m); 8.33 (2H, d, *J* = 10 Hz); 8.53 (1H, s); 12.04 (1H, s) (S8 Fig). ^13^C-NMR (50 MHz, DMSO-*d*_*6*_) δ 22.5; 28.8; 124.1; 124.8; 128.0; 128.4; 129.2; 130.1; 137.0; 140.8; 141.4; 145.0; 147.8; 163.5 (S9 Fig). IR (KBr) cm^-1^: 1314 e 1519 (ʋ N-O); 1652 (ʋ C = O), 3231 (ʋ N-H). % of purity >99% by HPLC

#### (*E*)-*N*-(4-boronic acid benzylidene)-5,6,7,8-tetrahydronaphthalene-2-carbohydrazide (2b, LASSBio-1763)

White yellow powder purified by recrystallization from ethanol, 91%; ^1^H-NMR (200 MHz, DMSO-*d*_*6*_): δ 1.74 (4H, sl); 2.76 (4H, sl); 7.20 (1H, d, *J* = 10 Hz); 7.69 (4H, m); 7.87 (2H, m); 8.44 (1H, s); 11.79 (1H, s) [S10 Fig]. ^13^C-NMR (50 MHz, DMSO-*d*_*6*_): δ 22.5; 28.8; 124.7; 126.0; 128.2; 129.0; 130.4; 134.5; 135.8; 136.8; 141.0; 147.5; 163.2 (S11 Fig). IR (KBr) cm^-1^: 1641 (ʋ C = O); 3231 (ʋ N-H); 3432 (ʋ O-H). % of purity >95% by HPLC

#### (*E*)-*N*-(4-boronic acid benzylidene)-naphthalene-2-carbohydrazide (2c, LASSBio-1760)

White powder purified by recrystallized from ethanol, 75%; ^1^H (200 MHz, DMSO-*d*_*6*_): δ 7.61–7.64 (2H, m); 7.73 (2H, d, *J* = 8 Hz); 7.90 (2H, d *J* = 8 Hz); 7.98–8.03 (4H, m); 8.20 (2H, s); 8.51 (1H, s); 8.56 (1H, s); 12.08 (1H, s) (S12 Fig). ^13^C-NMR (50 MHz, DMSO-*d*_*6*_): δ 124.3; 126.1; 126.9; 127.7; 127.9; 128.0; 128.1; 128.9; 130.7; 132.1; 134.3; 134.5; 135.7; 147.9; 163.3 [S13 Fig]. IR (KBr) cm^-1^: 3232–3311 (ʋ O-H), 1615 (ʋ C = O). % of purity >98% by HPLC

### Determination of aqueous solubility

The solubility test was made on the basis of ultraviolet (UV) absorbance obtained. The sweep wavelength was determined by greater absorbance characteristic of each compound. One saturated aqueous solution was stirred for 2 hours at 37°C, then filtered and the supernatant was transferred to a quartz cuvette to perform the reading. The solubility was determined by linear regression equation of the line. To obtain the line of points (known concentrations), it was necessary to use the absorbance obtained with progressive concentrations obtained by the dilution of a stock solution (1 mg/50 mL) in a solvent in which the analyzed compound present better profile solubility. Obviously, the choice of solvent can’t be absorbed by UV. For all compounds was used methanol. Then, it was made the chart with the dilutions and the absorbance, and was extracted from the line graph of the equation. Then, the straight line equation was solved where "Y" is the absorbance of the saturated solution in water, and the value "X" to be found will be the solubility of the compound [15].

### Pharmacology

#### Animals

The experimental groups were composed of 5–8 Swiss *Webster* mice (20–25 g) donated by the Animal Production Centre of the Instituto Vital Brazil (Niteroi, Rio de Janeiro). Animals (in a total of 208 mice) were maintained with a 12-h light/dark cycle and controlled temperature, ad libitum access to water and food. To avoid interference of food on absorption of substances administered to animals, they were fasted for 3 hours before the experiments. Animals were acclimatized to the laboratory for at least 1 hour before testing and were used only once throughout the experiments. After assays animals were euthanized with an overdose of choral hydrate. The experimental protocols used in this work followed the rules advocated by Law 11,794, of October 8, 2008 by the National Council of Animal Experimentation Control (CONCEA) and were approved by the Ethics Committee of Animal Use (CEUA), Science Center Health/UFRJ and received the number DFBCICB015-04/16.

#### Preparation and administration of compounds

All compounds tested were prepared in a stock solution at 100 μmol/mL of dimethyl sulfoxide (DMSO) and stored at -20°C until assays. The compounds were given orally at doses of 0.3, 3, 10 and 30 mg/kg, in a final volume of 100 μL of vehicle (Polysorbate 80). The selective and reversible inhibitor of IKKβ inhibitor, SC-514 [34] was given orally at a single dose of 10 mg/kg. The standard anti-inflammatory drug used was dexamethasone (2.5 mg/kg, i.p.).

#### Subcutaneous Air Pouch (SAP) model

The procedure used was similar to the first described method [35] with some modifications [36]. The SAP was formed on the back of the animals by injecting 10 mL of sterile air. After 3 days the cavity was injected over 7 mL of sterile air. At day 6, animals were orally treated with compounds and 60 minutes later, a sterile 1% carrageenan injection was performed into the formed cavity. A negative control group was treated with vehicle (Polysorbate 80) 60 minutes before receiving the injection of sterile carrageenan solution at SAP and a positive control groups received dexamethasone (2.5 mg/kg, i.p.) or SC-514 (10 mg/kg, p.o.). After 24 hours of carrageenan injection, animals were euthanized by an overdose of ketamine/xylazine, SAP was washed with 1 ml sterile phosphate buffer saline (PBS) and exudate was collected. Total leukocyte counts were determined in an automatic cell counter (CellPoch-100iV Diff, Sysmex). The exudates were centrifuged at 1,000 rpm, 10 minutes, at 4°C and aliquots of supernatant was stored at -20°C for subsequent measurements.

For the white blood cells counting, the mice were anesthetized with anesthetic ketamine/xylazine and 100 μL of blood were collected and placed into tubes with 15 μL of ethylenediamine tetraacetic acid (EDTA). For leukocyte count in the bone marrow, the femur was removed; its ends were cut and washed with 1 mL of sterile PBS. The white blood cells determination was performed in the automatic cell counter (Poch-100iV Diff, Sysmex).

#### Measurement of nitric oxide (NO)

The NO produced in the SAP supernatant was quantified according to the technique of the conversion of nitrate to nitrite [31]. The SAP samples were deproteinized and then admixed to a sample of sodium phosphate (0.5 M, pH 7.2), ammonium formate (2.4 M, pH 7.2), and *E*. *coli*. After incubation for 2 hours at 37°C, centrifugation was performed at 10,000 rpm for 10 minutes. Equal portions of the supernatant and Griess reagent were incubated for 10 minutes [37] and the absorbance was measured spectrophotometrically at 540 nm. Nitrate concentration values are expressed in μM, calculated from a standard curve of sodium nitrate done previously.

#### TNF-α measurement

The quantification of TNF-α was held at the SAP exudate. Specific ELISA kits (B&D ELISA OptEIA^™^) were used and TNF-α and their concentrations were determined according to the manufacturer's recommendations (B&D Biosciences).

#### Determination of reactive oxygen species (ROS) production

In order to study whether *N*-acylhydrazone were able to contain oxidative metabolism of neutrophils and investigate further if this is one of their mechanisms of action, leukocytes collected in SAP were placed in tubes (10^6^ cells) in a volume of 1 mL. Then incubation was performed at 37°C and 5% CO_2_ for one hour. Then the compounds were added at concentrations of 10 or 30 μM and incubated for 30 minutes at 37°C and 5% CO_2_. Cells were treated with 10 nM phorbol myristate acetate (PMA) and incubated for 45 minutes at 37°C and 5% CO_2_. 2'-7'diclorodihidrofluoescein diacetate (DCF-DA, 2 mM) was added to follow by further incubation for 30 minutes at 37°C. The emitted fluorescence was captured in the FL-1 channel flow cytometer (BD Accuri™) and was expressed as DCF-DA fluorescence [38].

#### Western blot analysis

Expression of the phosphorylated NF-κB p65 subunit was assessed by Western blot analysis. The monoclonal anti phospho-NFkB p65 (Ser536) and anti-β-actin antibodies (Cell Signaling, MA, USA) were used at 1:1,000 dilution for detecting the phosphorylated form of NF-kB and β-actin by Western blot analysis. Murine RAW 264.7 macrophages were seeded at 4 x 10^6^ cells/mL in 12 well plates. Cell lysates were prepared after 2 h incubation with 1 μg/ml of LPS (Lipopolysaccharide) and 1, 10 or 30 μM of LASSBio-1524, LASSBio-1760, LASSBio-1763 and LASSBio-1764. A control-unstimulated group was incubated with the compounds. Protein concentration was determined using a BCA Protein Assay Reagent kit (Thermo Fisher Scientific, Inc.). Proteins (30 μg) were separated by electrophoresis on 10% SDS-PAGE gel and then electrophoretically transferred to a PVDF membrane (Bio-Rad). The membrane was incubated with the antibodies during overnight, at 10°C. Immunoreactive bands were visualized using an enhanced chemiluminiscence system (ECL) substrate in a ChemiDoc Imaging system (Bio-Rad) [39].

### Statistical analysis

In vivo experiments were composed of 6–8 animals per group, randomly selected. For in vitro assays, each experimental group was done in triplicate. Each protocol was repeated at least 3 times. ED_50_ doses were calculated using GraphPad Prism 5.0 software and the following formula "Dose-response curves—Inhibition" and 95% confidence intervals. Y = 100/(1+10^((LogIC50-X)*HillSlope))).

Results are presented as mean ± standard deviation (SD). Statistical significance was calculated by analysis of variance (ANOVA) followed by Bonferroni post-test. P values less than 0.05 (* p <0.05) were considered significant.

## Supporting Information

S1 Fig Structure of IKK inhibitor SC-514. (TIF)

S2 Fig Total leukocytes on blood and bone marrow. The animals were pretreated orally 60 minutes before carrageenan injection in the SAP, with vehicle (Polysorbate 80) or compounds at doses of 30 mg/kg. Results expressed as mean ± standard deviation of the number of total leukocytes (x 10³/ μL). Statistical significance (p <0.05) was calculated by analysis of variance (ANOVA) followed by Bonferroni post-test.
(TIF)

S3 Fig Effect of the compounds cytotoxicity in Raw 264.7 cells. Cells were incubated in 96-well plates (10^5^ cells/well) were first incubated with and without indicated concentrations of the compounds for 2 hours, and then incubated with LPS (1 μg/mL) for 24 hours. Negative control without LPS treatment. Each value is expressed as mean± SD in triplicate experiments. Statistical significance (p <0.05) was calculated by analysis of variance (ANOVA) followed by Bonferroni post-test.
(TIF)

S4 Fig ^1^H-NMR spectrum of methyl 5,6,7,8-tetrahydronaphthalene-2-carboxylate (4a) (200 MHz, DMSO-*d*_*6*_). (TIF)

S5 Fig ^13^C-NMR spectrum of methyl 5,6,7,8-tetrahydronaphthalene-2-carboxylate (4a) (50 MHz, DMSO-*d*_*6*_). (TIF)

S6 Fig ^1^H-NMR spectrum of 5,6,7,8-tetrahydronaphthalene-2-carbohydrazide (5b) (200 MHz, CDCl_3_). (TIF)

S7 Fig ^13^C-NMR spectrum of 5,6,7,8-tetrahydronaphthalene-2-carbohydrazide (5b) (50 MHz, CDCl_3_). (TIF)

S8 Fig ^1^H-NMR spectrum of LASSBio-1764 (2a) (200 MHz, DMSO-*d*_*6*_). (TIF)

S9 Fig ^13^C-NMR spectrum of LASSBio-1764 (2a) (50 MHz, DMSO-*d*_*6*_). (TIF)

S10 Fig ^1^H-NMR spectrum of LASSBio-1763 (2b) (200 MHz, DMSO-*d*_*6*_). (TIF)

S11 Fig ^13^C-NMR spectrum of LASSBio-1763 (2b) (50 MHz, DMSO-*d*_*6*_). (TIF)

S12 Fig ^1^H-NMR spectrum of LASSBio-1760 (2c) (200 MHz, DMSO-*d*_*6*_). (TIF)

S13 Fig ^13^C-NMR spectrum of LASSBio-1760 (2c) (50 MHz, DMSO-*d*_*6*_). (TIF)
