# Supplementary figures and images for: Discovery of Novel Orally Active Tetrahydro-Naphthyl-N-Acylhydrazones with In Vivo Anti-TNF-α Effect and Remarkable Anti-Inflammatory Properties
Source: PLoS One. 2016 May 26;11(5):e0156271. doi: 10.1371/journal.pone.0156271 (PMC4881893; doi:10.1371/journal.pone.0156271)

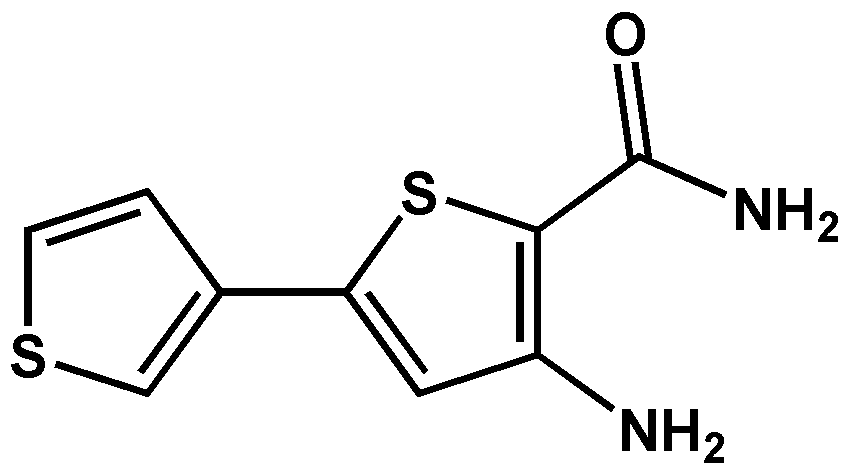

Supplement: S1 Fig — (TIF) [file pone.0156271.s001.tif]

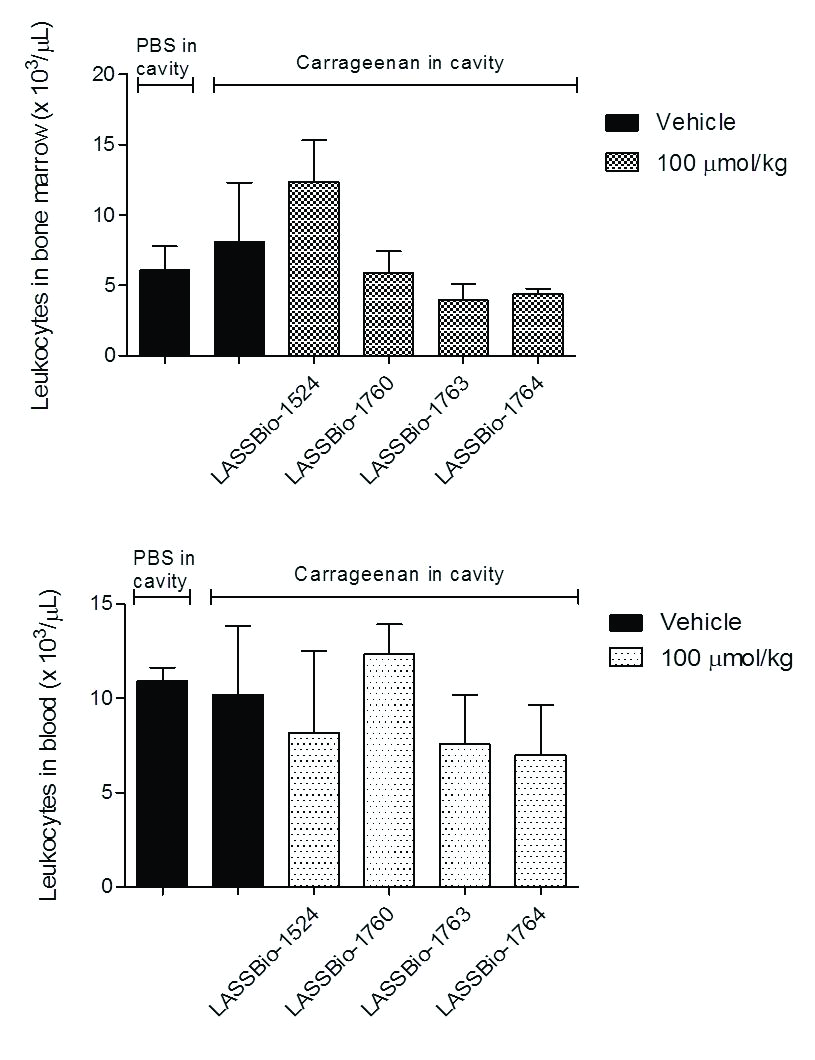

Supplement: S2 Fig — The animals were pretreated orally 60 minutes before carrageenan injection in the SAP, with vehicle (Polysorbate 80) or compounds at doses of 30 mg/kg. Results expressed as mean ± standard deviation of the number of total leukocytes (x 10³/ μL). Statistical significance (p <0.05) was calculated by analysis of variance (ANOVA) followed by Bonferroni post-test. (TIF) [file pone.0156271.s002.tif]

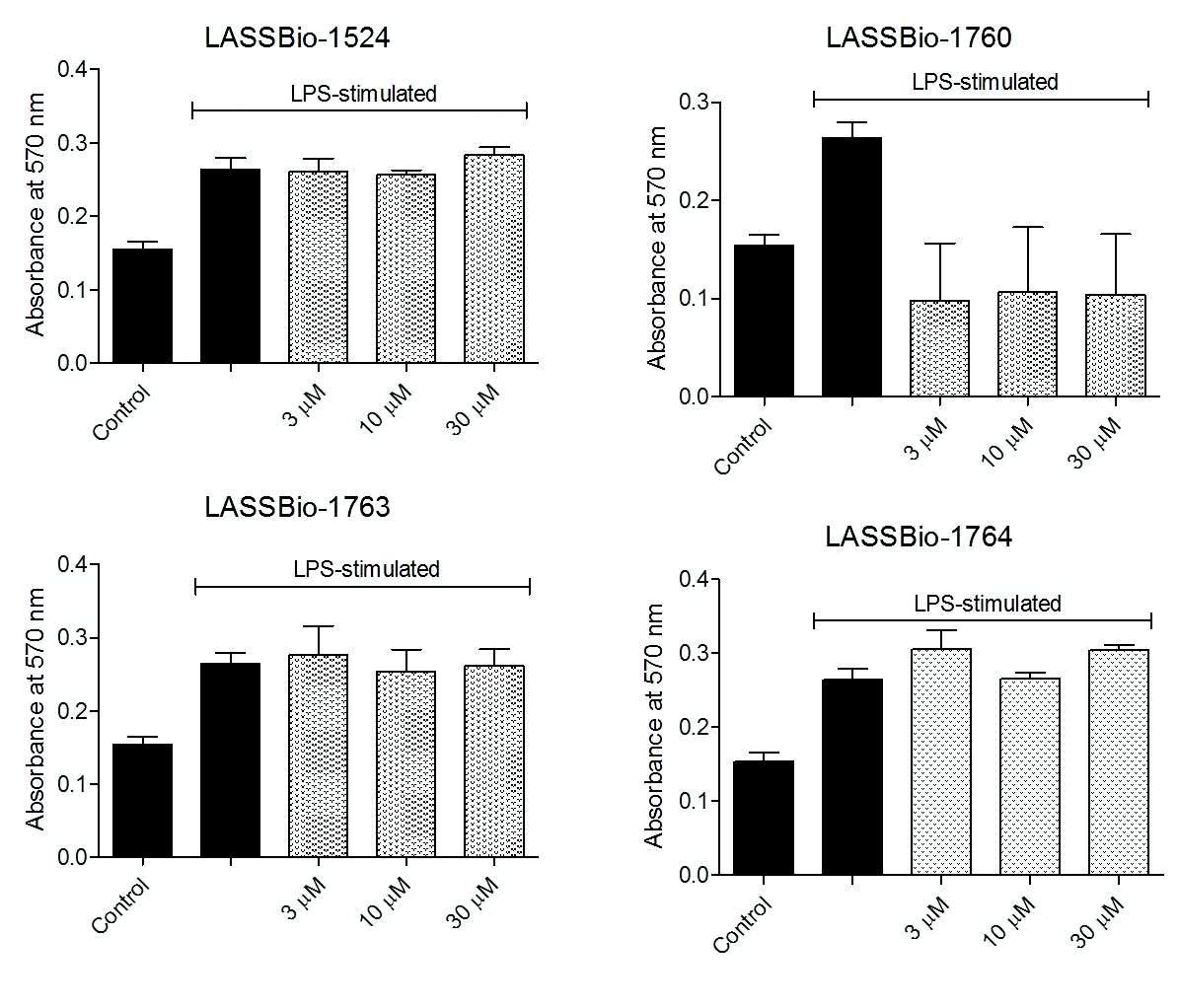

Supplement: S3 Fig — Cells were incubated in 96-well plates (105 cells/well) were first incubated with and without indicated concentrations of the compounds for 2 hours, and then incubated with LPS (1 μg/mL) for 24 hours. Negative control without LPS treatment. Each value is expressed as mean± SD in triplicate experiments. Statistical significance (p <0.05) was calculated by analysis of variance (ANOVA) followed by Bonferroni post-test. (TIF) [file pone.0156271.s003.tif]

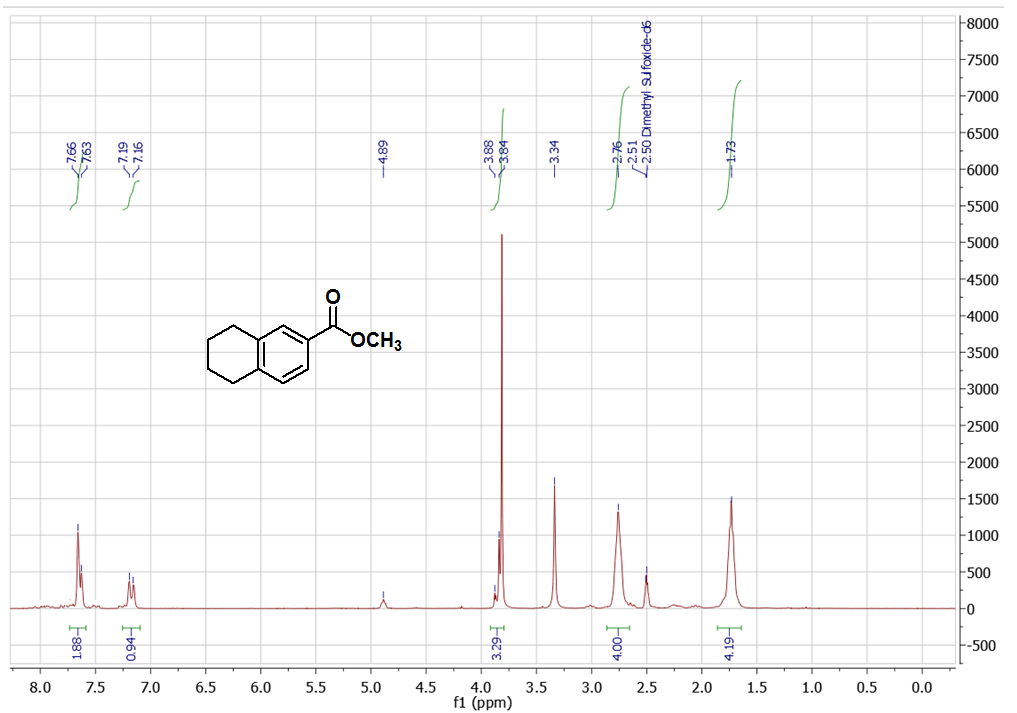

Supplement: S4 Fig — (TIF) [file pone.0156271.s004.tif]

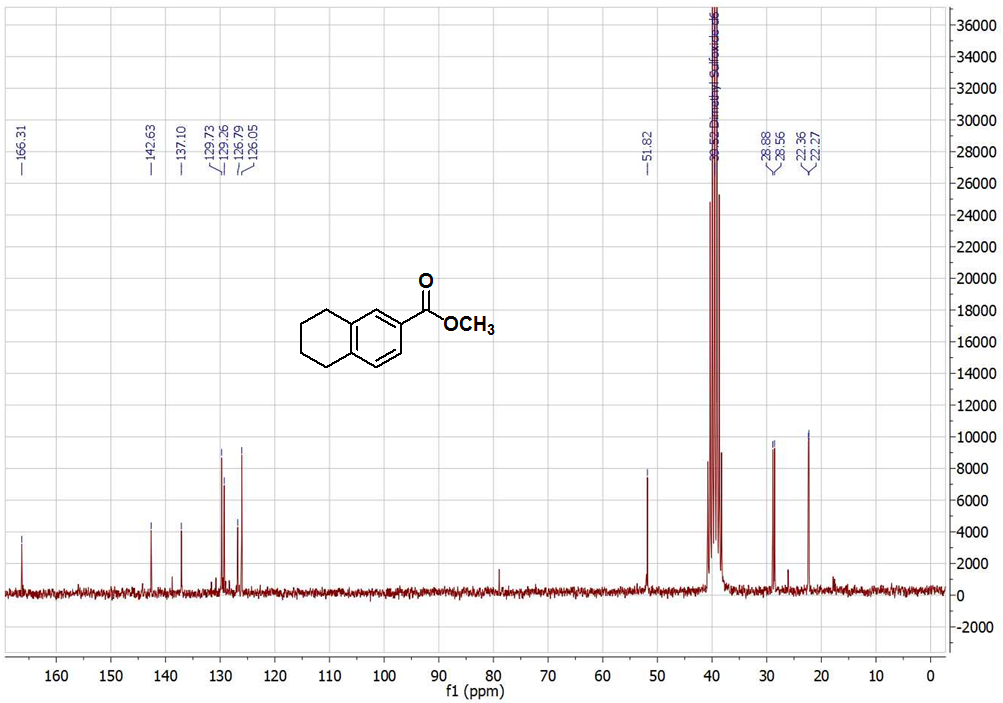

Supplement: S5 Fig — (TIF) [file pone.0156271.s005.tif]

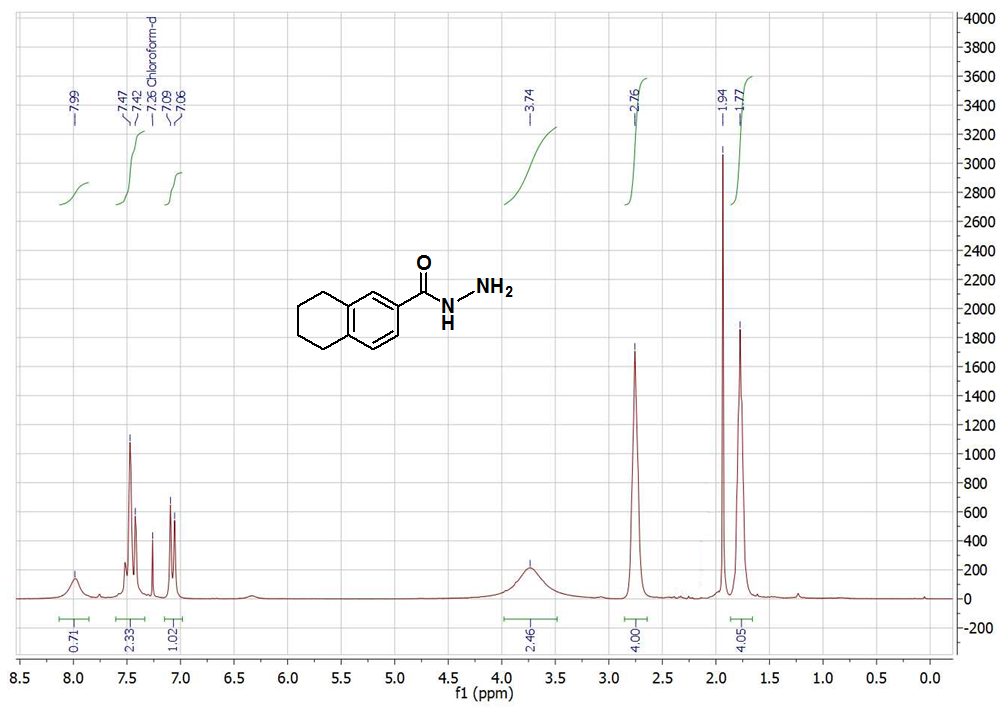

Supplement: S6 Fig — (TIF) [file pone.0156271.s006.tif]

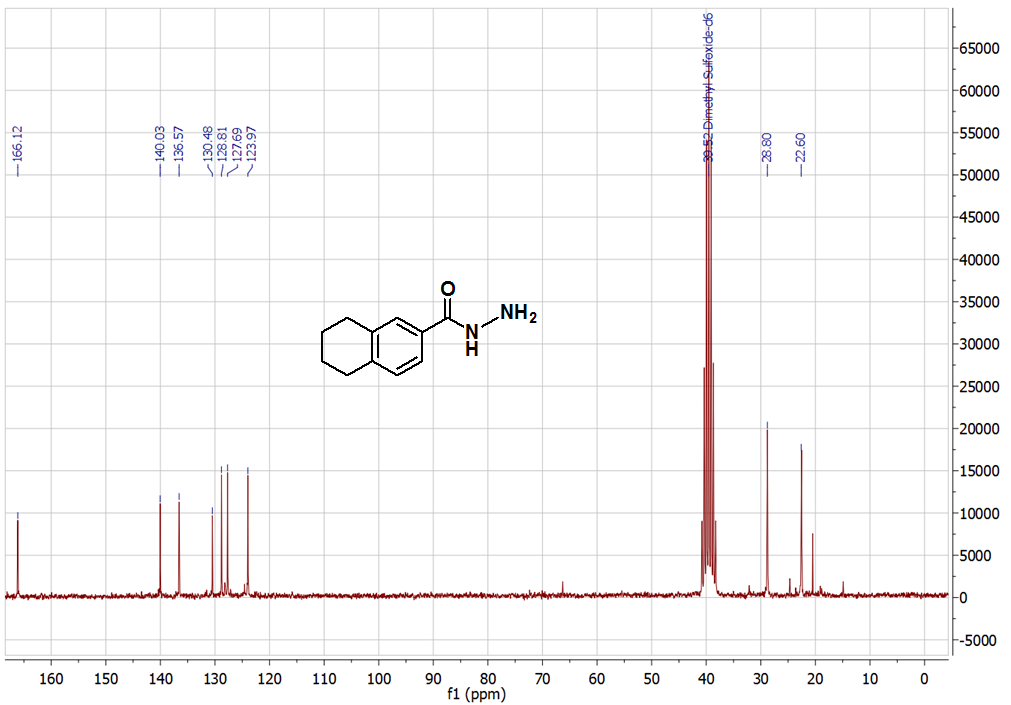

Supplement: S7 Fig — (TIF) [file pone.0156271.s007.tif]

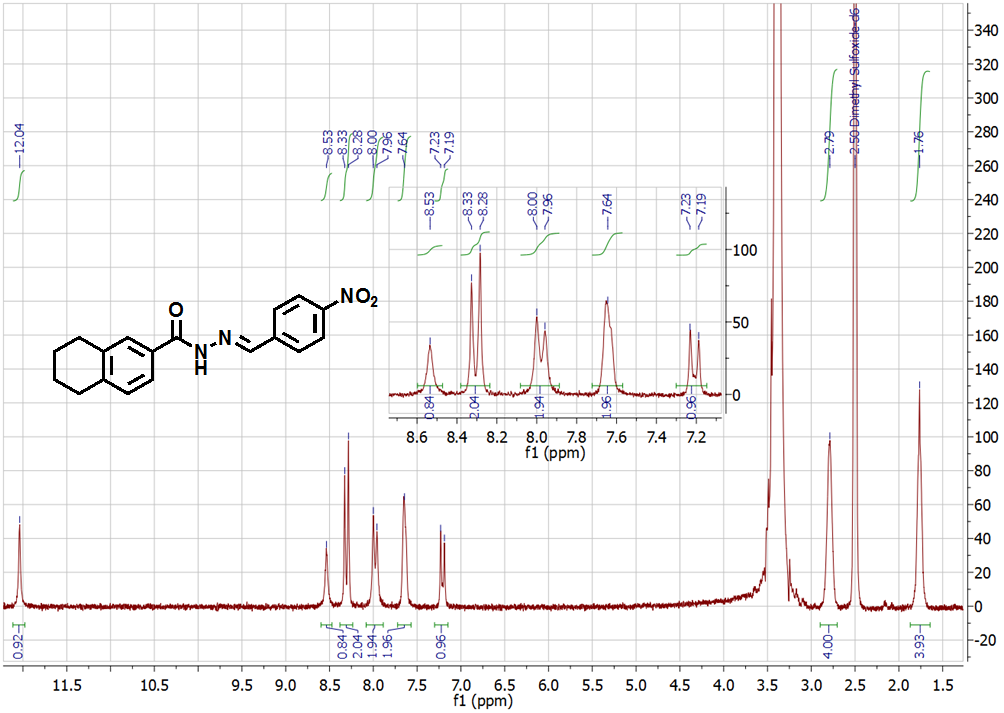

Supplement: S8 Fig — (TIF) [file pone.0156271.s008.tif]

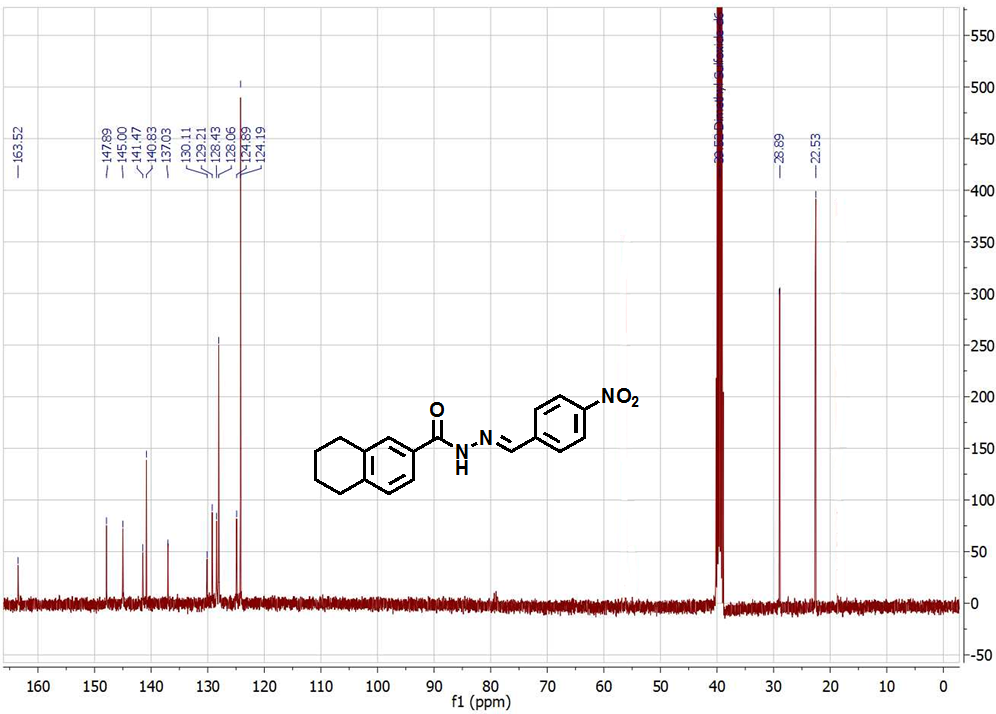

Supplement: S9 Fig — (TIF) [file pone.0156271.s009.tif]

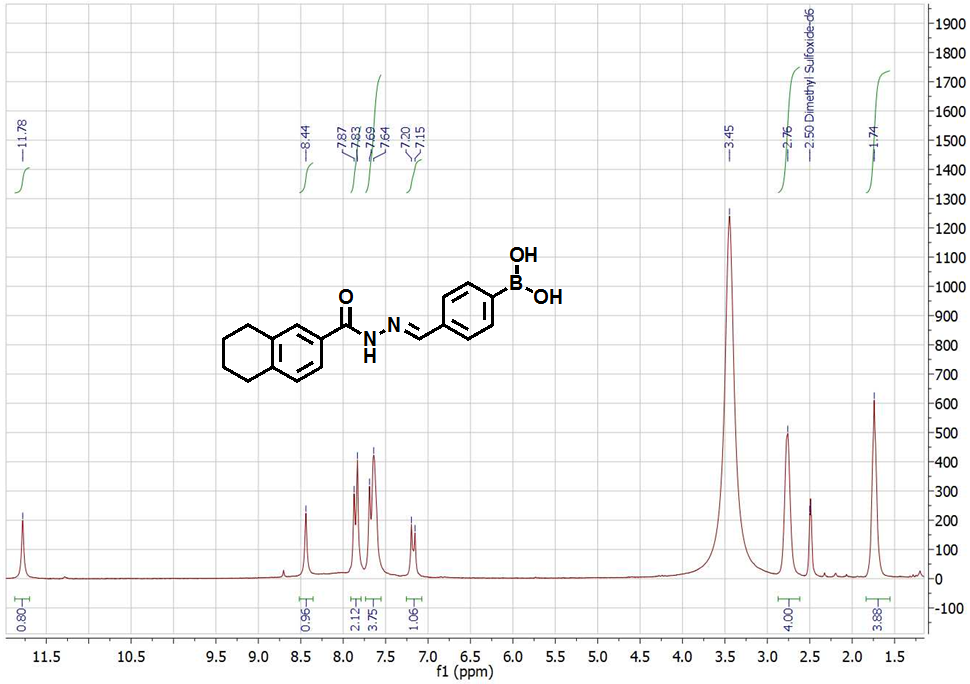

Supplement: S10 Fig — (TIF) [file pone.0156271.s010.tif]

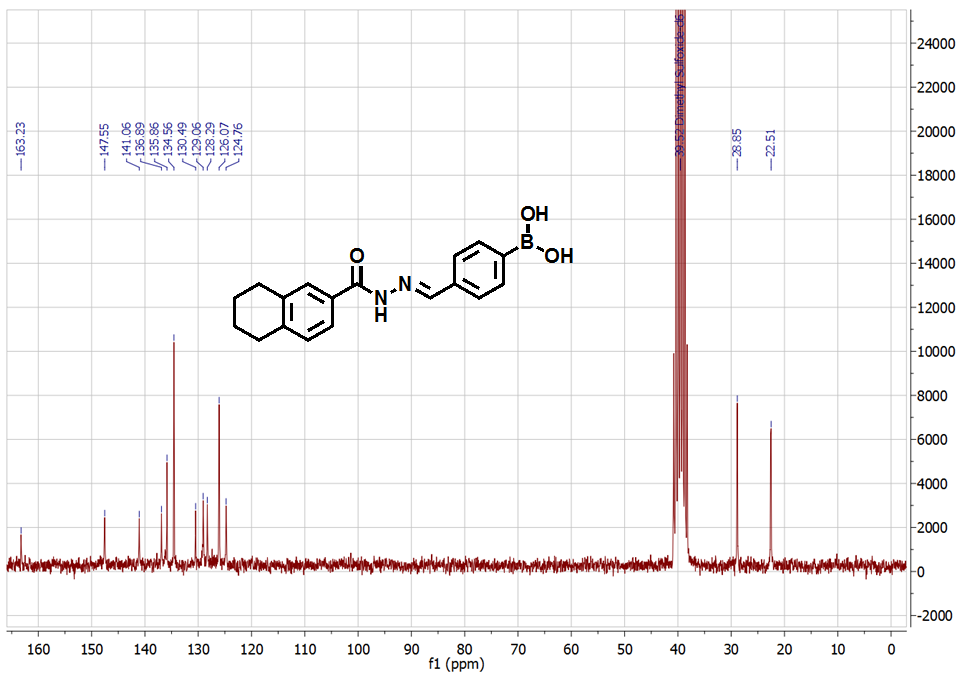

Supplement: S11 Fig — (TIF) [file pone.0156271.s011.tif]

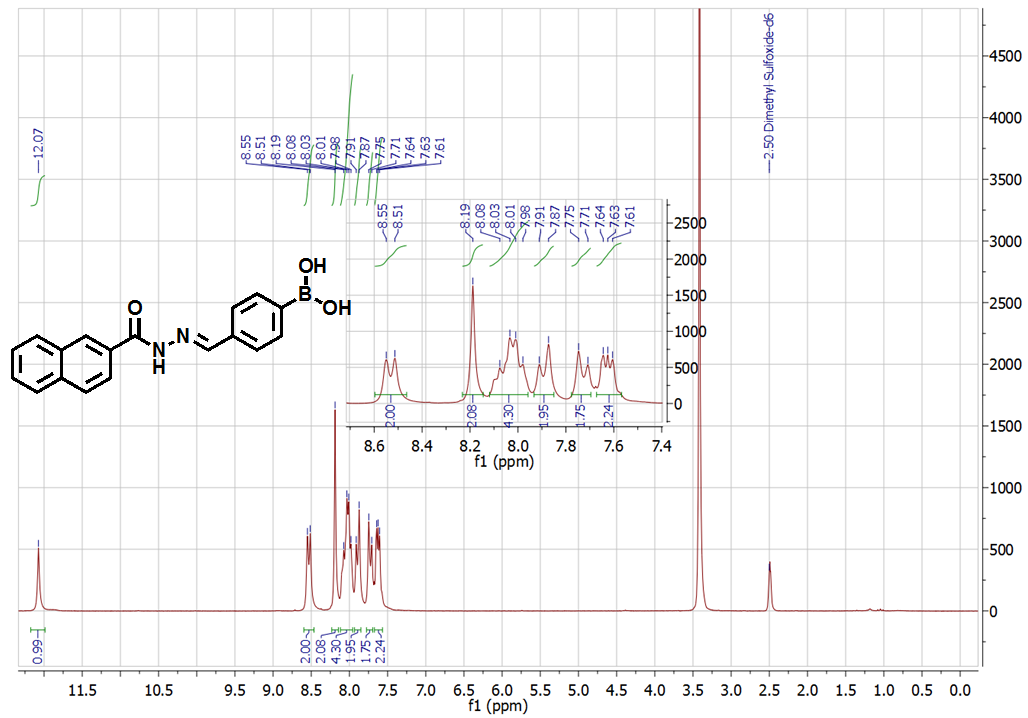

Supplement: S12 Fig — (TIF) [file pone.0156271.s012.tif]

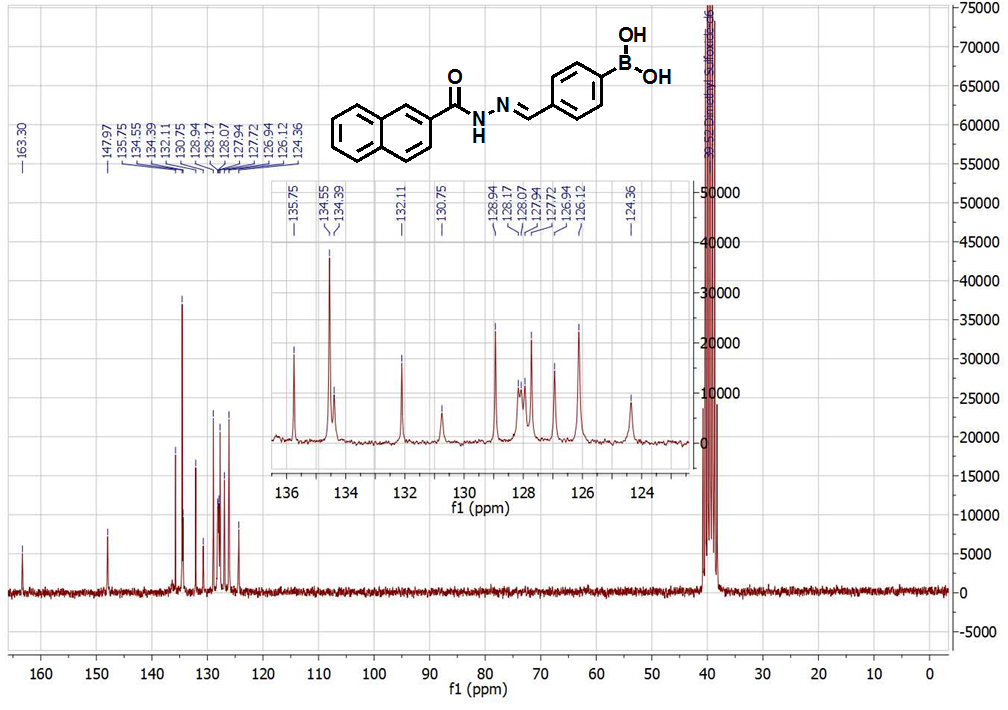

Supplement: S13 Fig — (TIF) [file pone.0156271.s013.tif]
